# Influence of climatic variables on maize grain yield and its components by adjusting the sowing date

**DOI:** 10.3389/fpls.2024.1411009

**Published:** 2024-06-27

**Authors:** Wenming Wu, Wei Yue, Jianjian Bi, Lin Zhang, Dafeng Xu, Chen Peng, Xiang Chen, Shiji Wang

**Affiliations:** ^1^ Crop Research Institute, Anhui Academy of Agricultural Sciences, Hefei, China; ^2^ Agricultural Meteorological Center, Anhui Meteorological Service, Hefei, China; ^3^ School of Agronomy, Anhui Agricultural University, Hefei, China

**Keywords:** maize yield, sowing date, kernel number per ear, climatic variables, high temperature

## Abstract

Yield and its components are greatly affected by climate change. Adjusting the sowing date is an effective way to alleviate adverse effects and adapt to climate change. Aiming to determine the optimal sowing date of summer maize and clarify the contribution of climatic variables to grain yield and its components, a consecutive 4-year field experiment was conducted from 2016 to 2019 with four sowing dates at 10-day intervals from 5 June to 5 July. Analysis of historical meteorological data showed that more solar radiation (SR) was distributed from early June to mid-August, and the maximum temperature (T_max_) > 32°C appeared from early July to late August, which advanced and lasted longer in 1991–2020 relative to 1981–1990. Additionally, the precipitation was mainly distributed from early June to late July. The climate change in the growing season of summer maize resulted in optimal sowing dates ranging from 5 June to 15 June, with higher yields and yield stability, mainly because of the higher kernel number per ear and 1,000-grain weight. The average contribution of kernel number per ear to grain yield was 58.7%, higher than that of 1,000-grain weight (41.3%). Variance partitioning analysis showed that SR in 15 days pre-silking to 15 days post-silking (SS) and silking to harvest (SH) stages significantly contributed to grain yield by 63.1% and 86.4%. The extreme growing degree days (EDD) > 32°C, SR, precipitation, and diurnal temperature range (DTR) contributed 20.6%, 22.9%, 14.5%, and 42.0% to kernel number per ear in the SS stage, respectively. Therefore, we concluded that the early sowing dates could gain high yield and yield stability due to the higher SR in the growing season. Meanwhile, due to the decreasing trend in SR and increasing T_max_ trend in this region, in the future, new maize varieties with high-temperature resistance, high light efficiency, shade tolerance, and medium-season traits need to be bred to adapt to climate change and increased grain yield.

## 1 Introduction

Maize is an important food crop and plays a crucial role in ensuring food security (Buriro et al., 2015; Seleiman et al., 2017; Dustgeer et al., 2021). However, as global warming and frequent occurrence of extreme weather, i.e., drought, heat damage, and wind, have increased across many of the world’s regions, it poses new challenges to food production (Huang et al., 2021; Roy et al., 2021; Seleiman et al., 2021; Xiao et al., 2022; Zafar et al., 2023). Anhui summer maize production region is located in the transitional zone between the Huang-Huai-Hai summer maize region and the Southeast spring maize region, and summer maize is normally grown after the harvest of winter wheat (Wu et al., 2023). Due to its particular geographical location and global warming, during the growing season of summer maize, waterlogging in the seedling stage and heat stress in the silking stage occur frequently, which resul in reduced grain yield (Liu et al., 2013; Wu et al., 2018; Shao et al., 2023).

Over the next two to three decades, the effects of climate change are expected to worsen, which needs the implementation of mitigation and adaptation strategies (Wang et al., 2023a). Currently, human management practices such as sustainable agriculture, irrigation, crop diversification, adjusting sowing date, increasing planting density, and technological advancements have been suggested more rapidly to mitigate and adapt to climate change worldwide (Challinor et al., 2016; Wang et al., 2021a; Srivastava et al., 2022; Yu et al., 2022; Wang et al., 2023b). Adjusting the sowing date is among the main low-cost adaptation strategies, and a modeling study promoted that the grain reduction due to global warming may only be partially offset by changes in phenology and sowing dates (Cirilo and Andrade, 1994; Chisanga et al., 2020; Bassu et al., 2021).

Variations in grain yield and its components according to different sowing dates have been documented by numerous studies (Tian et al., 2019; Zhu et al., 2022). Li et al. (2022a) found that in the North China Plain, high yield and yield stability could be obtained under the sowing dates in the early to mid-June due to more photosynthetically active radiation and the reduction of high-temperature days in the silking stage. Maize sowed on 10 June in Xinxiang also gained the highest grain yield (Zhou et al., 2016). However, another study showed that in the North China Plain, the highest average yields were obtained with the sowing dates of April, and the lowest yield was recorded on 13 June (Gao et al., 2021). Grain yield is significantly correlated with kernel number per ear and 1,000-grain weight. Adjusting the sowing date can optimize the planting window, optimally utilize available solar radiation, and adjust crop phenological stages according to the period when climate conditions are more suitable for growth, and avoid harmful stress events related to heat and water (Tsimba et al., 2013; Yang et al., 2021), which, in turn, significantly affect kernel number per ear and 1,000-grain weight. Kernel number is greatly determined during ~15-day pre-anthesis and ~15-day post-anthesis (Borrás and Vitantonio-Mazzini, 2018) because heat stress mainly occurs during this stage. Meanwhile, drought and heat stress at flowering also highly affect the grain-filling stage, reducing grain yield (Dong et al., 2021; Liu et al., 2022). Moreover, adjusting sowing dates also resulted in a variation of solar radiation during the growing season of maize, altering the photosynthesis product formation (Zhu et al., 2022). Apart from temperature and solar radiation, other climate factors, i.e., precipitation and wind, also significantly impact grain yield (Senthilkumar et al., 2015; Kaur et al., 2020).

Climate and agronomy, not genetics, gained high maize yield in favorable environments (Rizzoa et al., 2022). Climate variability accounted for roughly a third of the observed yield variability. In China, the value was 42% (Ray et al., 2015). Adjusting the sowing date could maximize climatic resources. Existing studies mainly focused on Northern China, and little is known about the transitional zone between the Huang-Huai-Hai summer maize region and the Southeast spring maize region, and the optimal sowing date is still under debate. Summer maize is commonly grown after winter wheat has been harvested in early June. However, the local farmer always postpones the sowing date until later than 25 June to alleviate the heat stress from mid-July to early August. Hence, field experiments of different sowing dates should be carried out to clarify the grain yield variation and determine the optimal sowing date in this region. In summary, in this study, a 4-year field experiment of different sowing dates was designed to clarify (1) the yield and yield stability and the contribution of grain components to grain yield under different sowing dates; (2) the contribution of climatic factors to grain yield and its components at crucial growth stages; and (3) the optimal sowing date under climatic change trend in the future.

## 2 Materials and methods

### 2.1 Experimental site

This 4-year field experiment was conducted at the experimental station of the Anhui Academy of Agricultural Sciences, China (31°57′ N, 117°11′ E), located in Hefei, Anhui province from June 2016 to October 2019. The soil type is yellow cinnamon soil. The upper 20-cm soil contained 20.6 g k^−1^ soil organic matter, 156.4 mg kg^−1^ alkali-hydrolyzable N, 21.1 mg kg^−1^ available P, 200.7 mg kg^−1^ available K, and a pH value of 6.7. The annual mean temperature was 16.0°C, and the annual mean precipitation was 1,000 mm.

### 2.2 Experimental design

Luyu 9105 was sown at a density of 6 plants m^−2^, as it is widely grown, high-yielding, resistant to multiple leaf diseases, i.e., *Bipolaris maydis* and rust disease, and highly adaptable. Four sowing dates were designed from 5 June to 5 July at 10-day intervals, i.e., 5 June, 15 June, 25 June, and 5 July. The plots were fully randomized, complete blocks with four replications. The size of each plot was 24.12 m^2^ (6.7-m length × 3.6-m width), with six rows and a row spacing of 60 cm.

The application rates of pure N, phosphorus, and potassium fertilizer in experimental sites were 240 kg ha^−1^, 105 kg ha^−1^, and 135 kg ha^−1^ using urea (containing N: 46%) and compound fertilizer (containing N, P_2_O_5,_ and K_2_O at 15:15:15); for the insufficient potassium fertilizer, we applied K_2_SO_4_ (containing K_2_O: 50%) as a supplement. All the fertilizers were applied to each plot prior to sowing. According to the actual situation in the field, the recommended dosages of chemical agents were used to control diseases, pests, and weeds.

### 2.3 Research methods

#### 2.3.1 Grain yield

At the physiology maturity stage, 30 ears in the middle three rows of each plot were selected to determine the yield and its components, i.e., the row number, kernel number per ear, and 1,000-kernel weight. One hundred kernels were sampled in the middle of each ear and weighed, then multiplied by 10 to determine the 1,000-grain weight. All the kernels were air-dried and the grain moisture content was determined according to the previous method to determine the final grain yield (Wu et al., 2023).


(1) $$\text{CV}=\frac{\text{standard deviation}}{\text{mean}}\times 100\%$$

#### 2.3.2 Growing seasons’ climatic variables analysis

The meteorological data, including daily mean temperature (T_mean_), maximum temperature (T_max_), precipitation, and sunshine hours, were obtained from the National Meteorological Data Center (http://data.cma.cn/).

The precipitation, solar radiation (SR), diurnal temperature range (DTR), and the extreme growing degree days (EDD) > 32°C were calculated in 15 days pre-silking to 15 days post-silking (SS) and silking to harvest (SH) stages, respectively. The SS stage was seen as the most critical period in the kernel setting in this study (Cicchino et al., 2010; Gao et al., 2021; Li et al., 2022a). According to a previous study, SR was calculated from sunshine hours (Wu et al., 2023). EDD is used to evaluate the risk of high temperature during the SS stage by summing maximum temperatures exceeding 32°C, and the calculation (seen in Equation 2) refers to Luo et al. (2023).


(2) $$\begin{array}{l} {\text{EDD}}_{32,\;t}=\sum_{t=1}^{N}{\text{DD}}_{t}\\ DD_{t}=\left\{\begin{array}{ll} 0 & if\;T_{mean}<32\\ T\text{mean}-30 & if\;T_{mean}\geq 32 \end{array}\right\} \end{array}$$

where *t* is the hourly time step, and *N* is the total number of hours in each growing phase. T_mean_ is the daily mean temperature.

#### 2.3.3 Statistical analysis

One-way ANOVA and Fisher’s least significant difference tests with p<0.05 (LSD0.05) were used to assess the effects of sowing date on the grain yield and its components by using SPSS 13.0 (SPSS, Chicago, IL, USA). Correlations were analyzed using Pearson’s correlation coefficient. The contributions of kernel number per ear and 1,000-grain weight to grain yield were calculated using the following equations, according to Liu et al. (2020).


(3) $$\begin{array}{l} a_{1}=\beta_{1}S_{x1}/S_{y}\\ b_{1}=\beta_{2}S_{x2}/S_{y} \end{array}$$

(4) $$\begin{array}{l} C_{KN}=[a_{1}/\left(a_{1}+b_{1}\right)]\times 100\%\\ C_{TGW}=[b_{1}/\left(a_{1}+b_{1}\right)]\times 100\% \end{array}$$

where *ɑ*_1_ and *b*_1_ are the standardized coefficients of kernel number per ear and 1,000-grain weight, *β*1 and *β*2 are the coefficients of kernel number per ear and 1,000-grain weight in the partial regression equation, and *S*_x1_ and *S*_x2_ are the standard deviations of kernel number per ear and 1,000-grain weight. *C*_KN_ and *C*_TGW_ are the contribution rates of kernel number per ear and 1000-grain weight to grain weight, respectively.

The effects of SR, EDD, DTR, and precipitation on the grain yield and its components in the SS and SH stages were analyzed by variance partitioning analysis (VPA) according to Li et al. (2024). The other data were analyzed using Excel 2016 (Redmond, WA, USA). The figures were drawn using SigmaPlot 10.0 (Systat, California, USA) and Origin 2021 (Northampton, Massachusetts, USA).

## 3 Results

### 3.1 Grain yield under different sowing dates

**Figure 1:**
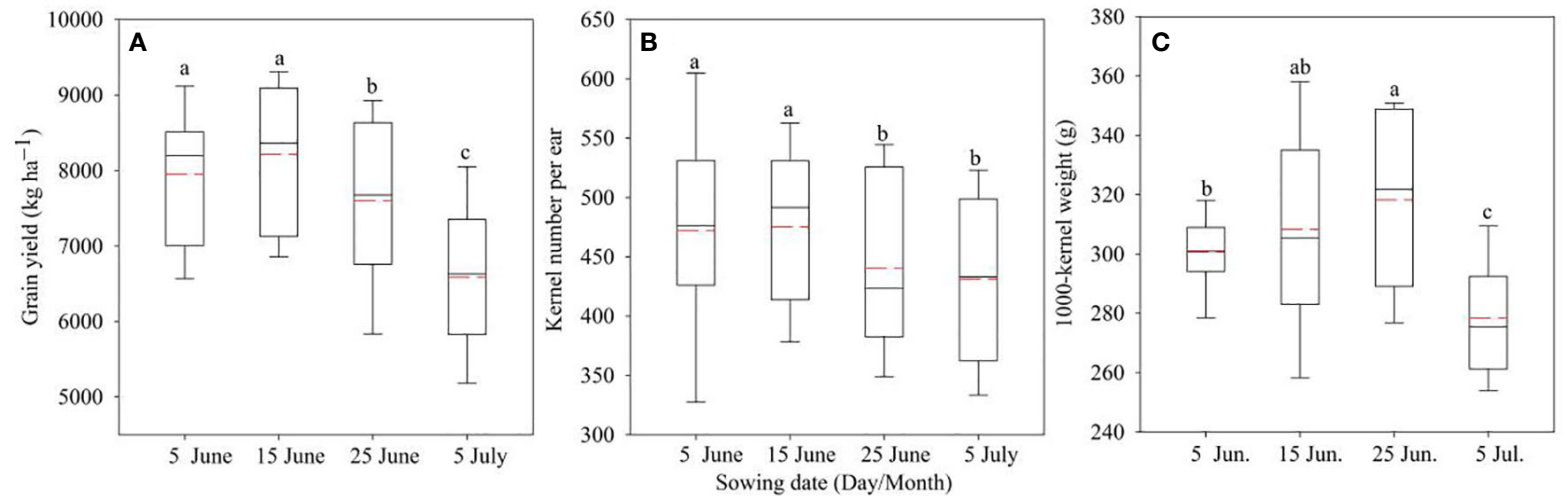
Grain yield and its components under different sowing dates. **(A)** represents grain yield, **(B)** represents kernel number per ear, and **(C)** represents 1,000-grain weight. Different letters above the boxes indicate significant differences between different sowing dates (P< 0.05). The red line represents the mean value, and the black line represents the median value.

Box plots for the grain yield, kernel number per ear, and 1,000-grain weight of different sowing dates are shown in **Figure 1**. The sowing date significantly impacted the grain yield and its components. The early sowing dates had significantly higher grain yield and kernel number per ear than the latter. However, the 1,000-grain weight showed a different trend. The highest grain yield and kernel number per ear were achieved with a sowing date of 15 June with an average grain yield of 8,215.2 kg ha^−1^. The lowest grain yield and components were obtained on 5 July, with a yield of 6,586.6 kg ha^−1^. The highest 1,000-grain weight was obtained with a sowing date of 25 June.

The intra-annual CV of grain yield of the four sowing dates ranged from 11.1% to 14.9%, and the lowest CV was obtained on 5 July (**Table 1**). Meanwhile, for kernel number per ear and 1,000-grain weight, the lowest CV was obtained on 15 June and 5 June, respectively.

**Table 1:** The intra-annual CV (%) of grain yield and its components under different sowing dates.

Sowing dates	Grain yield	Kernel number per ear	1,000-grain weight
5 June	11.12	18.91	4.11
15 June	11.81	13.81	10.70
25 June	14.07	16.45	8.97
5 July	14.92	16.06	7.07

### 3.2 Contribution of kernel number per ear and 1,000-grain weight to grain yield

**Figure 2:**
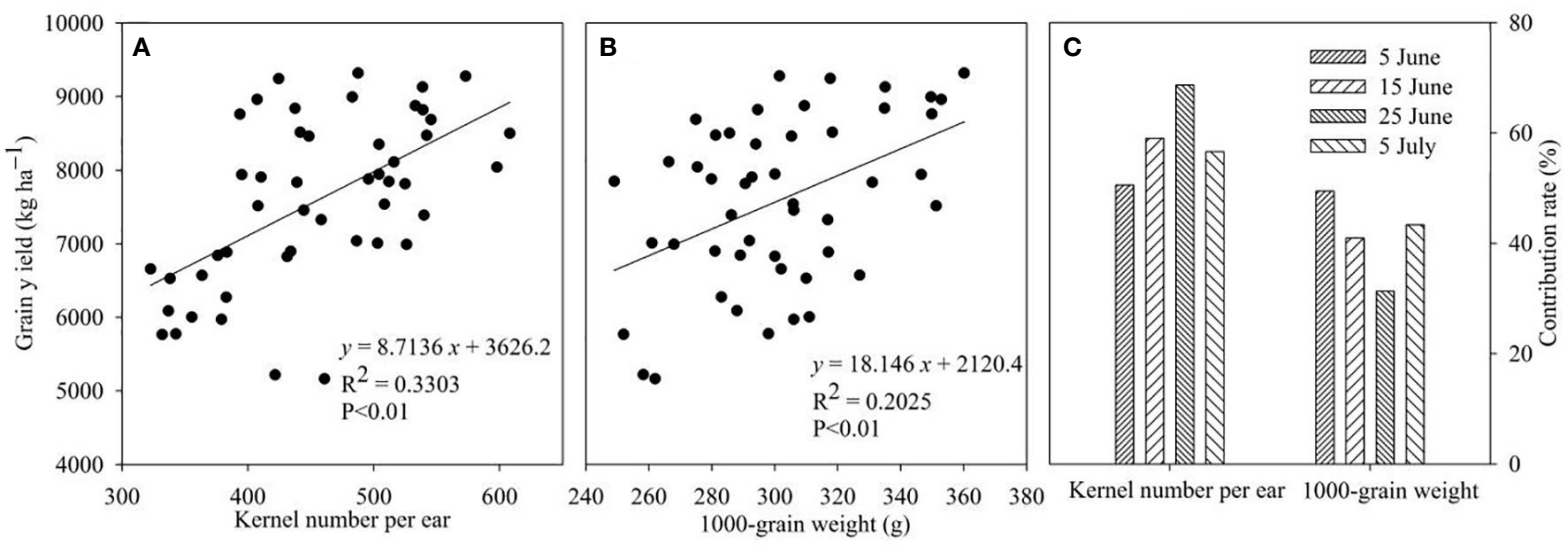
Relationship between grain yield and kernel number per ear **(A)**, 1,000-grain weight **(B)**, and the contribution of kernel number per ear and 1,000-grain weight to grain yield under different sowing dates **(C)**.

Regression analysis was performed, and the results indicated that kernel number per ear and 1,000-grain weight presented an apparent positive correlation with grain weight (**Figures 2A, B**).

Accordingly, to verify which component was the main factor that affected the grain yield, we calculated the contribution of kernel number per ear and 1,000-grain weight to grain yield, respectively (**Figure 2C**). The results showed that the contributions of kernel number per ear to grain yield of different sowing dates were 50.5%, 59.0%, 68.7%, and 56.6%, with an average value of 58.7%. With the postponement of sowing dates, the contributions of kernel number per ear initially increased, reaching the maximum at the sowing date of 25 June, and then decreased. However, the contribution of 1,000-grain weight to grain yield was lower than kernel number per ear, and the values were 49.5%, 41.0%, 31.4%, and 43.4%, respectively. Meanwhile, with the postponement of sowing dates, the contribution of 1,000-grain weight initially decreased, reaching a minimum on 25 June, then increased, which differed from the kernel number per ear.

### 3.3 Relationship between climatic variables and grain yield and its components

To clarify the relationship between climatic variables and grain yield and its components, VPA analysis between SR, EDD, DTR, and precipitation with grain yield and its components was conducted in the SS and SH stages, respectively. The regression equations are shown in **Table 2**. The results indicated that grain yield was positively affected by SR in the SS and SH stages, the kernel number per ear was positively affected by DTR, SR, and precipitation in the SS stage, and SR in the SH stage, while it was negatively affected by EDD in the SS stage. The 1,000-grain weight was positively affected by EDD in the SS stage and SR in the SH stage. However, it was negatively affected by DTR and precipitation in the SS stage.

**Table 2:** Multi-linear equation between grain yield, its components and climatic variables.

Stages	Dependent variables (*y*)	Multi-linear regression equation	R^2^	P
SS	Grain yield	*y* = -2.256 *x_1_* +10.351 *x_2_*-76.091 *x_3_* +2.785 *x_4_* +2940.349	0.474	<0.05
	Kernel number per ear	*y* = 0.166 *x_1_* +0.473 *x_2_* +73.144 *x_3_*-1.042 *x_4_*-355.798	0.689	<0.05
	1,000-grain weight	*y* = -0.136 *x_1_* +0.094 *x_2_*-29.110 *x_3_* +0.456 *x_4_* +483.489	0.346	<0.05
SH	Grain yield	*y* = 0.084 *x_1_* +12.559 *x_2_*-112.215 *x_3_* +1933.795	0.619	<0.05
	Kernel number per ear	*y* = 0.053 *x_1_* +0.466 *x_2_* +14.537 *x_3_* +77.697	0.448	<0.05
	1,000-grain weight	*y* = 0.043 *x_1_* +0.155 *x_2_*-5.771 *x_3_* +264.768	0.054	>0.05

y represents yield, kernel number per ear, and 1,000-grain weight; x_1_ represents precipitation; x_2_ represents SR; x_3_ represents DTR; and x_4_ represents EDD.

**Figure 3:**
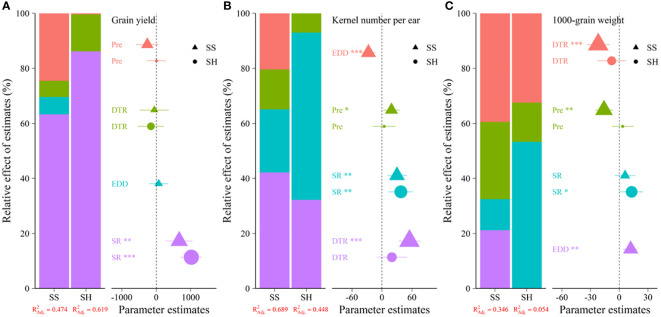
The relative effect of different climatic variables (SR, EDD, DTR, and precipitation) on grain yield and its components in the SS and SH stages. **(A)** represents grain yield, **(B)** represents kernel number per ear, and **(C)** represents 1,000-kernel weight. (P values are expressed as *** P< 0.001, ** P< 0.01, * P< 0.05).

VPA analysis showed that in the SS and SH stages, SR had the strongest independent explanatory power (63.1%, 86.4%) among the four climatic variables to grain yield, which was the main factor influencing grain yield (**Figure 3A**). EDD had 6.7% of grain yield, while precipitation and DTR had 24.7%, 5.5%, and 0.6%, 13.0%, respectively, in the SS and SH stages. While for kernel number per ear, DTR in the SS stage had the strongest explanatory power (42.0%), precipitation and SR had 14.5% and 22.9% of kernel number per ear, while EDD had 20.6% of it (**Figure 3B**). In the SH stage, SR had 60.8% of kernel number per ear. Hence, the results showed that EDD in the SS stage was the primary climatic variable that decreased the kernel number per ear. As for the 1,000-grain weight, EDD showed a positive explanatory power (21.4%), different from kernel number per ear in the SS stage, and DTR and precipitation had 39.2% and 28.5% (**Figure 3C**). In the SH stage, SR had 53.1% of it.

### 3.4 Climatic variables distribution

**Figure 4:**
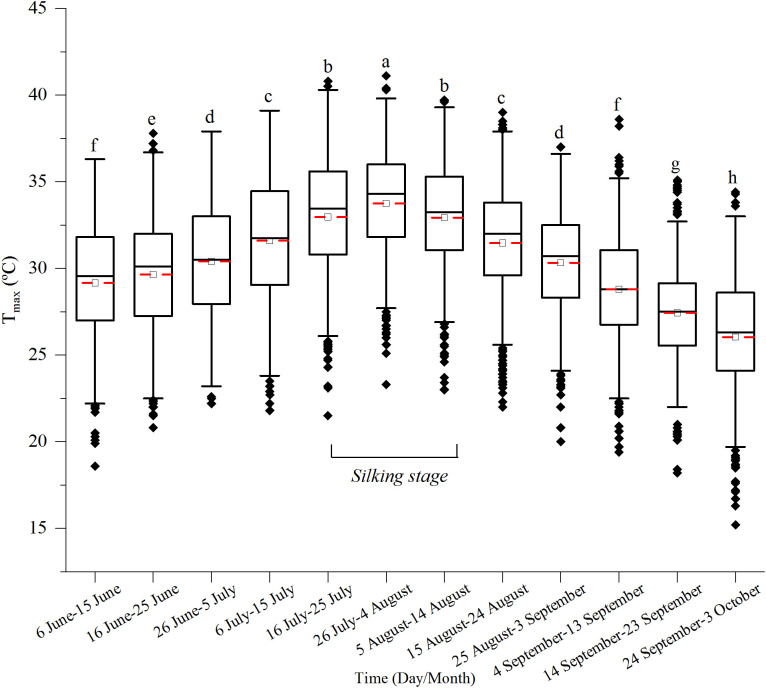
Distribution of T_max_ during the growing season of summer maize from 1981 to 2020. Different letters above the boxes indicate significant differences between different durations at 10-day intervals (P< 0.05). The red line represents the mean T_max_, and the black line represents the median T_max_. Black diamonds are abnormal values.

**Figure 5:**
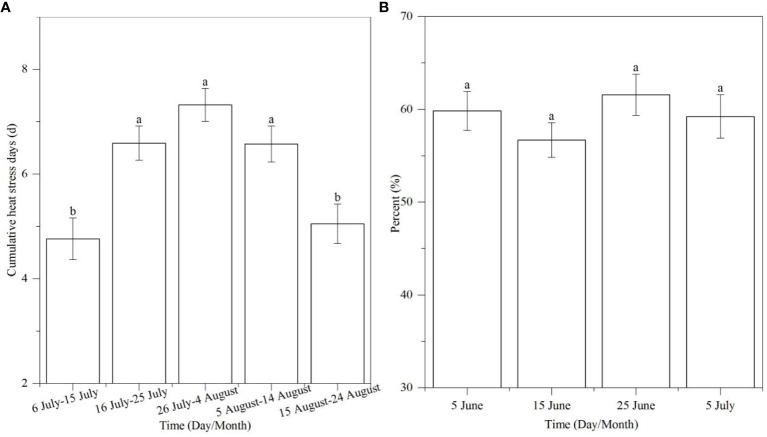
The cumulative heat stress days of different durations at 10-day intervals **(A)** and the occurrence percent of T_max_ > 32°C of different sowing dates in the SS stage **(B)**. Different letters above the columns indicate significant differences between durations at 10-day intervals and sowing dates (P< 0.05).

To predict the climate change and the sowing date, we calculated the T_max_, precipitation, and SR for 40 years at 10-day intervals from 1 June to 3 October. Results showed that T_max_ > 32°C occurred from 6 July to 24 August, which coincided with the SS stage of experimental sowing dates (**Figure 4**). The cumulative heat stress days > 32°C reached the maximum from 26 July to 4 August, then decreased, and the values of cumulative heat stress days from 16 July to 14 August at 10-day intervals were significantly higher than other dates (**Figure 5A**). Additionally, we calculated the percent of heat stress days during the SS stage of different sowing dates; the results showed that there was no significant difference in heat stress days between different sowing dates, which indicated that when only considering T_max_, adjusting the sowing date could not avoid the high heat stress (**Figure 5B**).

The quadratic equation was used to simulate the changing trends of the T_max_ at 10-year intervals from 1981 to 2020 (**Table 3**). Results showed that the distribution T_max_ > 32°C occurred in 53–60 days after 1 June in 1981–1990, 41–80 days in 1991–2000, 31–79 days in 2001–2010, and 32–84 days in 2011–2020, which indicated that the occurrence of T_max_ advanced and lasted longer from 1980 to 2020.

**Table 3:** The quadratic equation of the T_max_ at 10-year intervals from 1981 to 2020.

Years	Simulated equation	R2	Range
1981–1990	*y* = -0.0020 *x* ^2^ + 0.2256 *x* + 25.676	0.8090	28 July–4 August (8)
1991–2000	*y* = -0.0018 *x* ^2^ + 0.2177 *x* + 26.110	0.7845	16 July–24 August (39)
2001–2010	*y* = -0.0015 *x* ^2^ + 0.1647 *x* + 28.298	0.8185	6 July–23 August (48)
2011–2020	*y* = -0.0019 *x* ^2^ + 0.2214 *x* + 26.882	0.7555	7 July–28 August (53)

y represents mean T_max_ at 10-year intervals from 1981 to 2020; x represents days after 1 June.

**Figure 6:**
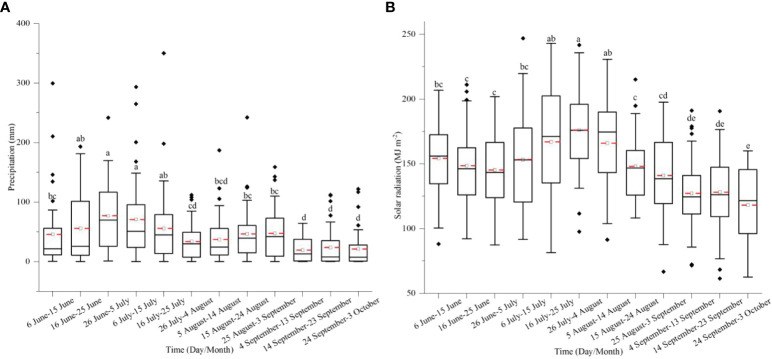
The precipitation **(A)** and SR **(B)** distribution during the growing season of summer maize from 1981 to 2020. Different letters above the boxes indicate significant differences between different durations at 10-day intervals (P< 0.05).

Precipitation distribution during the growing season initially increased and reached the maximum from 26 June to 5 July, then decreased. The amount of precipitation from 16 June to 25 July was significantly higher than other dates, and the maximum precipitation occurred from 26 June to 5 July (**Figure 6A**). The SR reached the maximum from 26 July to 4 Aug, which differed from precipitation (**Figure 6B**).

**Figure 7:**
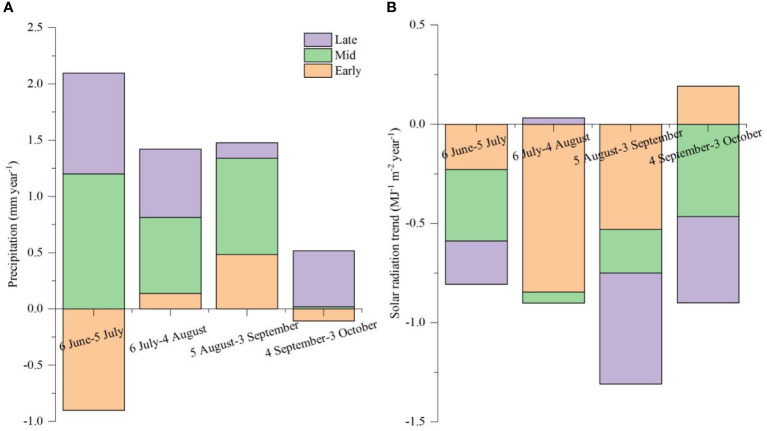
Changing trends of precipitation **(A)** and SR **(B)** in the growing season of summer maize from 1981 to 2020. The trend was calculated as expressed by the slopes of linear regressions between years and precipitation or SR every 10-day (different colors in the bars). The calculation referred to Li et al. (2021).

Different from T_max_, precipitation showed no significant change from 1981 to 2020, but the SR decreased with the year increase (**Figure 7A**). Moreover, the change in precipitation at 10-day intervals showed an increasing trend from mid-June, which differed from the SR. During the growing season of maize, the SR changing at 10-day intervals showed a decreasing trend (**Figure 7B**).

## 4 Discussion

### 4.1 Optimal sowing date and effect of climatic variables on grain yield

The distinct change in climatic conditions during the growth period of summer maize thereby influences its growth sensitivity and yield response (Wang et al., 2022; Li et al., 2022b). Previous studies have demonstrated that adjusting sowing dates can influence crop growth rates and the duration of phenological phases, subsequently affecting the potential grain yield and its components (Bonelli et al., 2016; Han et al., 2022; Rizzoa et al., 2022). Our results indicated that sowing dates ranging from 5 June to 15 June could obtain higher a grain yield and yield stability than other sowing dates, which may be due to the early dates having higher kernel number per ear. The kernel number per ear was the main factor affecting the grain yield at a given sowing density. As the sowing date was postponed to 25 June, even though the 1,000-grain weight reached the maximum, still, it could not compensate for the reduction of kernel number per ear and resulted in a reduction of grain yield, which was different from the finding from Bonelli et al. (2016).

A reasonable sowing date could obtain and optimize the utilization of climatic resources (Li et al., 2022b). As analyzed in **Figure 3**, climatic variables in the SS and SH stages exhibited a significant correlation with grain yield and its components. Among the climatic variables, SR strongly correlated with grain yield and its components, particularly in the SH stage. With the postponement of sowing dates, the values of SR in the SS (565.1 MJ m^−2^, 543.4 MJ m^−2^, 501.7 MJ m^−2^, and 480.9 MJ m^−2^) and SH (580.6 MJ m^−2^, 539.8 MJ m^−2^, 519.4 MJ m^−2^, and 466.8 MJ m^−2^) stages significantly decreased. Increasing SR around the SS and grain-filling stages of early sowing dates were associated with higher grain yield and its components, which was consistent with the finding from Yang et al. (2019). They concluded that when SR during grain filling was less than 623 MJ m^−2^, increases in maize yield were primarily related to the amount of SR (Yang et al., 2019), which may be due to SR driving crop growth and has a positive correlation with biomass (Yan et al., 2017; Liu et al., 2021; Yang et al., 2021). Hou et al. (2021) noted that a decrease of 100 MJ m^−2^ in SR would also reduce 0.85 t ha^−1^ in maize grain yield in China.

Apart from SR, our results indicated that the contributions of EDD and precipitation to grain yield were lower than that of SR, which was different from the findings in the Huang-Huai-Hai summer maize region and North China Plain (Chen et al., 2012; Gao et al., 2018; Wang et al., 2022). They concluded that the sensitivity of summer maize to precipitation and temperature was more substantial than that of SR in the SS stage but more sensitive to temperature and SR in the later growth stages. The difference may be owing to the high precipitation from June to July in our study, which caused the decrease in SR (**Figure 3A**). Different from grain yield, VPA analysis showed that EDD negatively affected the kernel number per ear and had a 20.6% contribution in the SS stage, DTR and precipitation positively affected it. Nevertheless, the 1,000-grain weight had a different trend, possibly due to the reduction of kernel number per ear. Kernel number per ear is primarily determined by the climatic conditions that occurred around the SS stage (Borrás and Vitantonio-Mazzini, 2018; Wang et al., 2023c). Heat stress decreased floret differentiation, induced pollination failure (Wang et al., 2021b). As illustrated in **Figure 4**, T_max_ > 32°C overlapped with the SS stage of all the experimental sowing dates, and the occurrence percent of T_max_ > 32°C during the SS stage had no significant difference among different sowing dates (**Figure 5B**). However, early sowing dates had higher a kernel number than the latter. These results may be caused by the higher precipitation, SR, and DTR during the SS stage of the early sowing dates, which may offset the negative effect of the high-temperature stress (**Figure 6A**). Previous studies indicated that irrigation would be an alternative approach to maintaining crop yield by alleviating heat stress through evaporative cooling (Li et al., 2020; Wu et al., 2020). Furthermore, the later sowing dates of summer maize increased T_max_ > 32°C exposure, and the increasing trend in the minimum temperature decreased DTR, which may reduce the duration of the vegetative period, in turn decreasing the dry matter accumulation and kernel number per ear (Bassu et al., 2014; Asseng et al., 2015; Gao et al., 2021). In summary, the climate change in the growing season of summer maize resulted in optimal sowing dates ranging from 5 June to 15 June, with higher yields and yield stability, mainly because of high SR in SS and SH stages. The adverse effect of heat stress could be mitigated by other climatic variables, which indicated that the selection of sowing dates should consider the compound effect of different climatic variables.

### 4.2 Prediction of future sowing dates under climate change

Our study showed that SR, temperature, and precipitation were the main climatic variables that impacted the grain yield of summer maize. Based on historical meteorological data, we analyzed the changing trends of SR, T_max_ > 32°C, and precipitation during the growing season of summer maize. The results indicated that the duration of T_max_ > 32°C significantly increased from 1991 to 2020 compared to that of 1981 to 1990. The occurrence time of T_max_ > 32°C mainly occurred between 7 July and 28 August from 2011 to 2020 and lasted 53 days, which shifted earlier and lasted longer in the past 40 years, and these results were consistent with the finding from Wang et al. (2018). The duration of T_max_ > 32°C overlapped the SS and grain-filling stages. High temperature around the SS stage destroyed pollens and damaged pollination by association with high EDD, which resulted in a reduction of kernel number and grain yield (Cicchino et al., 2010; Lobell et al., 2012; Wang et al., 2018). Thus, the sowing date should be adjusted to avoid the high-temperature period around the critical stage. In the North China Plain, a previous study showed that postponing the sowing date could reduce the probability of suffering high temperatures, and summer maize could obtain more light resources during the grain-filling stage due to the increasing SR in September (Tian et al., 2019; Li et al., 2022b). However, in order not to interrupt the normal sowing of winter wheat, the summer maize in the transitional zone between the Huang-Huai-Hai summer maize region and the Southeast spring maize region was sown after the harvest of winter wheat from June to July; no matter how the sowing dates changed, the SS stage was concentrated from late July to mid-August, and for which it was impossible to avoid the period of high-temperature occurrence. Furthermore, during the growing season of summer maize, SR in July and August was significantly higher than in September (**Figure 6B**), and in the past 40 years, SR has shown a decreasing trend, especially in the later growing season (**Figure 7B**). Therefore, the sowing date should be advanced before 15 June to obtain abundant SR. Other research in soybean also indicated that a delayed sowing date to 20 June in the Huang-Huai-Hai region decreased temperature exposure and growing degree days, reducing the duration of grain filling, leaf area index, and photosynthesis, in turn decreasing the number of grains per plant and 1,000-grain weight (Zheng et al., 2024). From a precipitation perspective, in the past 40 years, precipitation has been mainly concentrated on 6 July and 25 July (**Figure 6A**), and exhibited an increasing trend from mid-June to early September, which may have resulted in drought in the sowing stage and waterlogging in the seedling stage for the early sowing date. Consequently, in the future, when the sowing date of summer maize in this region advances before mid-June, farm irrigation facility should be constructed to mitigate the drought stress in the sowing period of an early sowing date. In conclusion, properly advancing the sowing date before 15 June could prolong the growing season and increase cumulative SR. However, under more severe warming and the sustained reduction of SR, new cultivars need to be bred to mitigate the more adverse effects of rising temperature and lower SR.

## 5 Conclusions

Sowing date significantly impacted grain yield and its components. The optimal sowing date of summer maize in the transitional zone between the Huang-Huai-Hai summer maize region and the southeast spring maize region ranged from 5 June to 15 June. VPA analysis showed that SR was the primary climatic variable influencing grain yield, contributing 63.1% and 86.4% of the total explanatory power in the SS and SH stages. Adjusting the sowing date could not avoid the heat stress around the SS stage, and EDD negatively decreased the kernel number per ear. However, early sowing dates could obtain sufficient SR and increase the grain yield. Under the background of increasing temperature, sustained reduction of SR, and increasing precipitation in this region, in the future, new cultivars need to be bred to suit the climatic change in this region, such as high-temperature resistance, high light efficiency, and shade tolerance to increase grain yield.

## Data availability statement

The original contributions presented in the study are included in the article/supplementary material. Further inquiries can be directed to the corresponding author.

## Author contributions

WW: Conceptualization, Data curation, Formal analysis, Funding acquisition, Investigation, Methodology, Project administration, Resources, Software, Supervision, Validation, Visualization, Writing – original draft, Writing – review & editing. WY: Data curation, Formal analysis, Methodology, Software, Writing – original draft. JB: Investigation, Writing – original draft. LZ: Conceptualization, Data curation, Formal analysis, Funding acquisition, Investigation, Writing – original draft. DX: Formal analysis, Funding acquisition, Methodology, Writing – original draft. CP: Investigation, Methodology, Writing – original draft. XC: Data curation, Methodology, Software, Writing – original draft. SW: Conceptualization, Data curation, Methodology, Supervision, Validation, Writing – original draft.
